# Innovative Coating–Etching Method of Biocarrier Fabrication for Treating Wastewater with a Low C/N Ratio

**DOI:** 10.3390/polym14153010

**Published:** 2022-07-25

**Authors:** Ning Yu, Daijun Zhang, Yu Lei, Jianhui Wang, Yang Dong, Youpeng Chen

**Affiliations:** 1 Chongqing Institute of Green and Intelligent Technology, Chinese Academy of Sciences, Chongqing 400714, China; zhangdaijun@cigit.ac.cn (D.Z.); dongyang@cigit.ac.cn (Y.D.); 2 University of Chinese Academy of Sciences, Beijing 100049, China; 3 National Research Base of Intelligent Manufacturing Service, Chongqing Technology and Business University, Chongqing 400067, China; jhwang@ctbu.edu.cn; 4 College of Environment and Ecology, Chongqing University, Chongqing 400044, China; ypchen@cqu.edu.cn

**Keywords:** biodegradable polymers, biofilm, nitrate removal, solid carbon source, wastewater treatment

## Abstract

A novel method was used to fabricate the bio-carrier with both a high specific surface area and good compatibility. The results of monitoring the growth of biofilms at a low C/N ratio (0.83) showed that resulting carrier-PLA-cavity offered certain advantages for biofilm growth by providing an appropriate microenvironment for bacterial growth in wastewater treatment. The biofilm on carrier-PLA-cavity grew and updated faster than the naked-carrier. The biomass and thickness of biofilms growing on carrier-PLA-cavity were 10 kg/m^3^ and 500 μm, respectively. From the wastewater tests, 90% of the total nitrogen was removed via simultaneous nitrification and denitrification (SND) by the biofilm biomass attached to carrier-PLA-cavity, compared to 68% for the naked-carrier. The COD removal efficiency values of the carrier-PLA-cavity and naked-carrier were 94% and 86%, respectively. The microbial community analysis of carrier biofilms showed that *Halomonas* was the most abundant genus, and heterotrophic nitrification and denitrification were responsible for nitrogen removal in both reactors. Notably, this method does not require any complicated equipment or structural design. This novel method might be a promising strategy for fabricating biocarriers for treating wastewater with a low C/N ratio.

## 1. Introduction

Wastewater treatment with biofilms is mostly based on activated sludge technology that removes nutrients from wastewater by micro-organic biofilms growing in/on carriers. The method of the biofilm process is low-cost and useful for wastewater treatment, but a shortage of carbon sources for denitrification limits the performance for treating wastewater with a low C/N ratio [1]. Biofilm carriers, as the key element of this technology, greatly affect the efficiency of wastewater treatment [2,3,4,5,6,7,8,9,10]. Therefore, reasonable selection in biocarriers is important. A large specific surface area and good compatibility of carrier material are the two crucial bases for biocarrier evaluation [11,12,13,14,15].

Various efforts have been made to increase the specific surface area of carriers, such as changing the formation of carriers with porous structures, adding textures with high length–diameter ratios, and grafting natural fibers and nanofillers with high mechanical properties [2,4,7,16,17,18,19,20]. For example, Szikora et al. [2] used granular solids with tiny pores to increase the specific surface area of biofilm carriers and discovered that biofilms on granular solids had more intensive dehydrogenase enzyme activity than those grown on normal solids. Maslon et al. [3] studied the removal of organics and nutrients from synthetic wastewater by a moving bed sequencing batch biofilm reactor using Bio-Ball^®^ carriers, which had a higher specific surface area and provided a better attachment medium than classical plastic carriers for the growth of microorganisms. Choi et al. [4] used activated carbon as a porous material to improve the specific surface area, which enhanced the stability of biological perchlorate reduction in fixed-bed biofilm reactors. An excellent effect of wastewater treatment by polymer fiber-based carriers with a high length–diameter ratio was reported by Tamas et al. [5]. The biofilm community grown on the fiber-based carrier with good colonization properties was dominated by polymer fiber-based carriers. Gapes et al. [6] compared two types of biocarriers with different structures in terms of their internal and external mass transfer resistance when a biofilm was present. Dong et al. designed and fabricated a fullerene-type biocarrier with a high specific surface area using a 3D printing technique [7]. The biofilms on the 3D-printed biocarriers exhibited higher microbial activity and stronger adhesion ability than traditional plastic carriers. The above literature is concerned with improving the sewage treatment effect by increasing the carrier surface area, but the compatibility between microorganisms and carriers has not been well-studied.

To improve the compatibility between microorganisms and carriers, it is important to choose a suitable carrier material with good biocompatibility. Some biodegradable polymer materials can be used as carriers, as they not only have good compatibility but also can be used as carbon sources for biological heterotrophic denitrification [1]. Such biodegradable polymers (BDPs) can be divided into two classes [21]: natural materials including starch and cross-linked starch [22] and synthetic polymers such as polycaprolactone (PCL; [1], poly(butylene succinate) (PBS; [23]) and polylactic acid (PLA; [24]). Advantages of natural materials lie in their low price and extensive sources [1,25]. However, due to the low mechanical properties of natural materials [26,27], synthetic polymers with enhanced biocompatibility have been used in wastewater treatment [28].

There are two ways to improve biofilm growth: one is to increase the specific surface area of the carrier, and the other is to improve the compatibility between the carrier and microorganism. However, for conventional methods of preparing biocarriers, such as surface modification and grafted BDP methods, there are no effective ways to increase both the quantity and efficiency of biofilm formation at the same time.

Therefore, in this study, we demonstrate a novel method to fabricate biocarriers with both a high specific surface area and good compatibility, denoted as the coating–etching method. BDPs are first wrapped on the surface of the biocarrier by the coating method, and then the biocarrier with the coating of BDPs is corroded by the chemical etching method. This method can improve both the biocompatibility and the specific surface area of the biofilm carrier, and facilitates the adhesion/growth and shedding of biofilms through the microporous BDP coating. In particular, these methods do not require any complicated equipment or structural design. This novel research has promising potential in the large-scale fabrication of carrier BDPs with micropore formation to improve the low C/N ratio of wastewater treatment performance.

## 2. Materials and Methods

### 2.1. Materials

The carrier was K3 (Texture: polyethylene, diameter: 30 mm, MBBR, MIER Co., Ltd., Wuxi, China). The biodegradable polymer was PLA (4032D, Nature works Co. Ltd., Blair, NE, USA). The dosage of PLA was 0.89% by mass to treat K3 per ton. NaOH and CH_2_Cl_2_ were purchased from Chemical Reagent Company, Tianjin, China. Activated sludge was acquired from Beibei Municipal Wastewater Plant (Chongqing, China). The physical and chemical properties of materials are shown in Table 1.

### 2.2. Two-Step Coating–Etching Method

The two-step coating–etching method was used to deposit BDP films with cavities. Initially, the PLA particles were dissolved in CH_2_Cl_2_ to prepare a PLA solution (30 g/L). Then, the carrier was immersed into the PLA solution for ~60 s to maintain sufficient interaction time of the carrier with the coating solution for complete wetting. In addition, the carrier was pulled upward at a constant speed (~2 mm/s). Then, a thin layer of PLA solution was entrained, which was named carrier-PLA. The excess liquid on the surface of carrier-PLA was drained from the surface. Ultimately, the carrier-PLA was immersed in NaOH solution (20 g/L) for 2 min at 50 °C. The microporous cavity formation of carrier-PLA was formed, which was denoted as carrier-PLA-cavity. After cooling to room temperature, the carrier-PLA-cavity was rinsed with water and dried at 60 °C for 15 min. The thickness of the PLA layer was controlled by the number of coatings. The size and density of the microporous cavity on the surface of carrier-PLA was adjusted by the concentration of NaOH solution.

### 2.3. Synthetic Wastewater

The synthetic wastewater was composed of four parts: substrates, salt, trace elements, and mineral media. The substrates were ammonium, nitrate, and chemical oxygen demand (COD). The compositions of the trace element solution and mineral media are shown in Table 2 and Table 3. The parameters of synthetic wastewater are shown in Table 4. The COD/TN ratio of the synthetic wastewater was 0.83.

### 2.4. Reactor Set-Ups and Operation

**Figure 1:**
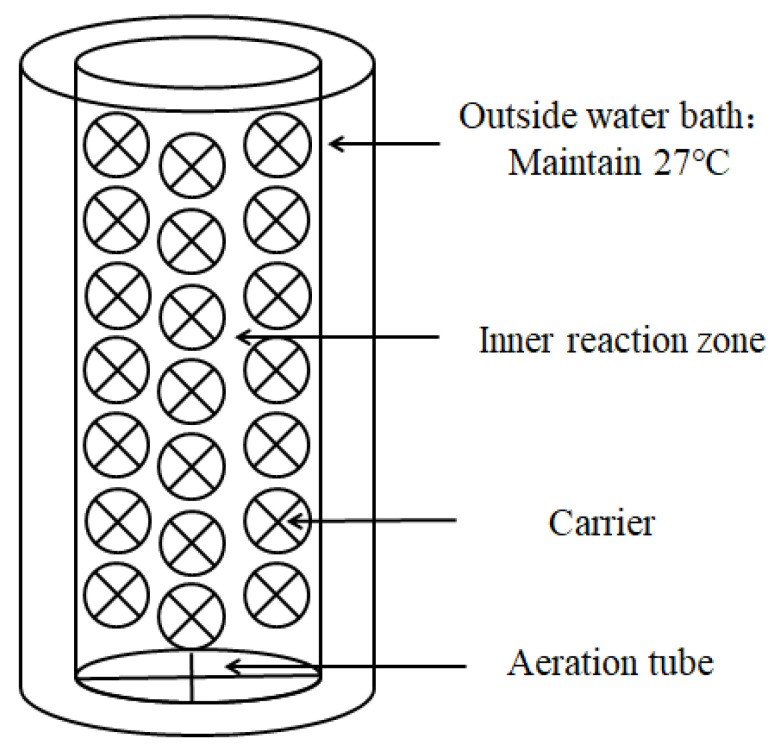
Schematic of the sequencing biofilm batch reactor.

A lab-scale batch reactor was constructed from commercially available Plexiglas vessels (size: 24 cm × 50 cm) with a working volume of 22.6 L [7]. It consisted of two parts (Figure 1). One was an outside water bath thermal insulation layer to maintain the temperature at 27 °C, and another one was an inner zone of reaction site. The carriers were suspended in the reaction zone and organized by lines.

The reactor was inoculated with activated sludge, which had a mixed liquor suspended solid (MLSS) content of 11.275 g/L and an SV (setting velocity) of 82. The mixed liquor from the secondary sedimentation tank was inoculated at a ratio of 1:3 (*v*/*v*) with reactor volume, and the reactor was operated with a designed synthetic feed to support biomass formation on the carriers [7]. After 48 h, the activated sludge was discharged, and simulated wastewater was added to the reactor. To maintain the stability of the experimental system, an aerobic metabolic mode was adopted, with a total cycle period of 8 h (retention time), consisting of a 15 min fill phase, 6.5 h aerobic reaction phase with recycling, 15 min out phase, and 1 h decant time [7].

### 2.5. Characterization of the Carrier and Biofilm

The surface of the carrier (carrier, carrier-PLA, and carrier-PLA- cavity) was observed by LEICA-DMLP light microscopy at 23 °C, and images (200× magnification) were collected with a CCD camera. The structure of the biofilm attached to the carriers was examined by scanning electron microscopy (SEM, JSM-7800F, JEOL, Tokyo, Japan) at an acceleration voltage of 5 kV. The scale bar was 100 μm. The working range was 100 nm~500 μm.

To evaluate the biomass supported by biocarriers, samples of biocarrier elements were taken from the reactor. Biofilm solids were determined by the difference in weight of dried biocarriers (105 °C for ≥ 1 h) before and after biofilm removal. Removal of biofilm solids was performed in NaOH through mechanical shaking and ultrasound treatment at 60 °C. All the parameters were measured according to standard methods [29].

A subset of the bio-carriers was selected for biofilm thickness measurements. Bio-carriers with attached biofilm were carefully cut into several sections, and biofilm thickness was measured with a pair of vernier calipers. On account of the thickness heterogeneity of the biofilm, 10 measurements were averaged to determine the biofilm thickness. The mass and thickness of the biofilm were directly measured and the PLA layer was ignored because it was too thin.

### 2.6. DNA Extraction and Microbial Community Analysis

Ten-gram biofilm samples were obtained from each reactor with stable water quality for DNA extraction and microbial community analysis. DNA was extracted from biofilms with the Power Soil DNA Kit (QIAGEN, Redwood City, CA, USA) according to the manufacturer’s instructions, and extracted DNA was quantified by a NanoVue plus Spectrophotometer (GE, Boston, MA, USA).

The bacterial community structure was assessed by Illumina PE300 sequencing at the Shanghai Majorbio Biopharm Biotechnology Co., Ltd. (Shanghai, China). Bacterial amplicon libraries were constructed for Illumina sequencing using the primers 338F (5′;- ACTCCTACGGGAGGCAGCAG-3′;) and 806R (5′;- GGACTACHVGGGTWTCTAAT-3′;) for the V3–V4 regions of the 16S rRNA gene [30]. All data processing was conducted using Mothurv.1.31.2 (East Lansing, MI, USA). To obtain high-quality sequences, the low-quality sequences were removed by eliminating those without exact matches to the forward primer, those without recognizable reverse primers, those with lengths shorter than 200 nucleotides, and those containing any ambiguous base calls (Ns). To create operational taxonomic units (OTUs), tags were aligned to the SILVER 111-compatible alignment database using the align.seqs command. The remaining sequences were then assessed for potential chimeras, and the chimeras were removed. The sequences were clustered into OTUs by setting a 0.03 or a 0.05 distance limit (equivalent to 97% or 95% similarity, respectively), and rarefaction data and Shannon and Chao diversity indices were generated for each sample. The sequences were phylogenetically assigned to taxonomic classifications using an RDP-naïve Bayesian rRNA classifier with a confidence threshold of 80% [30]. The sequences obtained through this process are listed in Table 4.

Based on the OTU information, rarefaction curves and alpha diversity indices referring to community diversity (Shannon and Simpson), community richness (Chao and Ace), and sequencing depth (Coverage) were calculated by Mothur. The 100% stacked column reflecting community structures at the phylum and genus levels was drawn by Origin 2018.

### 2.7. Analytical Method for Wastewater

The performance of the reactor was assessed by monitoring COD and total nitrogen (TN), i.e., NH_4_-N, NO_3_^−^-N, and nitrite (NO_2_-N), throughout the operation. Both of these parameters were analyzed according to standard methods for the examination of wastewater [29]. All experiments were conducted in duplicate, and average values were used for data analysis.

## 3. Results and Discussion

### 3.1. Structure of PLA Films by Light Microscopy

**Figure 2:**
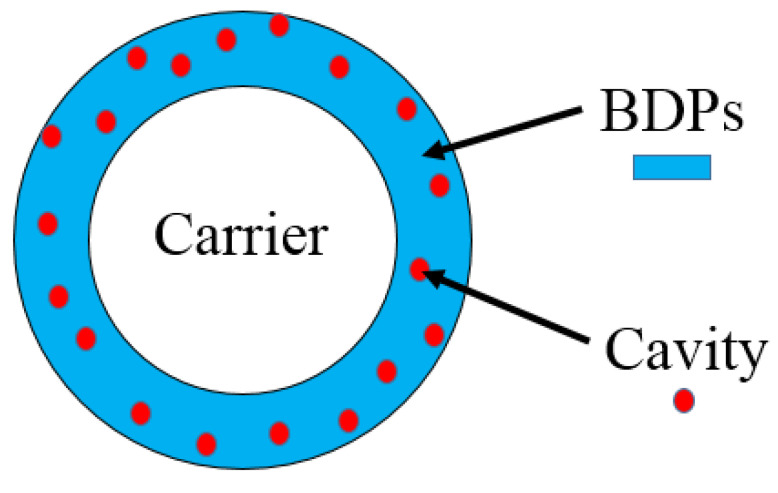
Schematic diagram of Carrier-PLA-Cavity.

A schematic diagram of carrier-PLA-cavity is shown in Figure 2. It has three parts: carrier, PLA film, and microporous cavity on the film.

**Figure 3:**
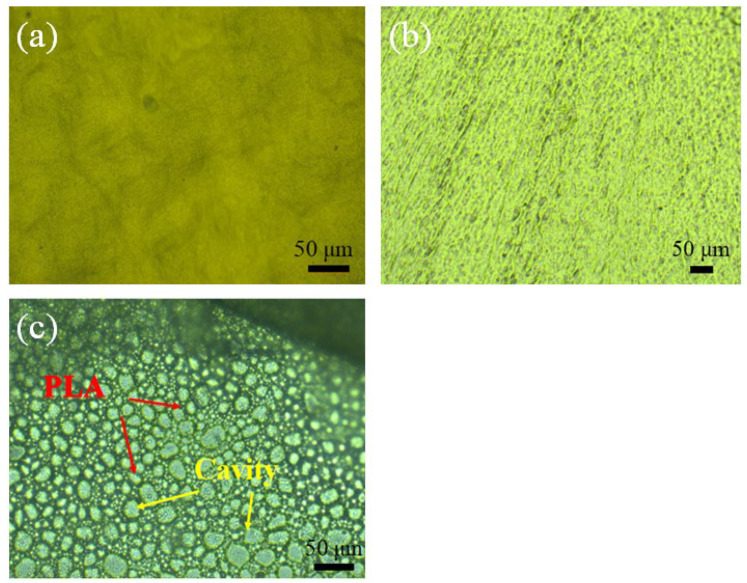
Surface appearance of samples observed: (**a**) naked-carrier, (**b**) carrier-PLA, and (**c**) carrier-PLA-cavity (optical microscopy, 200× magnification).

A PLA film with a microporous cavity was coated on the surface of the carrier. The structure of the PLA film was observed by light microscopy with and without the etching treatment process. The surface of the naked-carrier was smooth (Figure 3a). After coating treatment, the naked-carrier was coated with a thin film of PLA (Figure 3b). The relative degree of embossment on the surface of carrier-PLA was larger than that of the naked-carrier after coating with the PLA film. Upon further etching processing, the islands-in-the-sea structure of the carrier-PLA-cavity was observed by the corrosion of NaOH to PLA molecules (Figure 3c). Therefore, the surface roughness of the carrier-PLA-cavity was higher than that of the naked-carrier.

### 3.2. Biofilm on the Carriers

**Figure 4:**
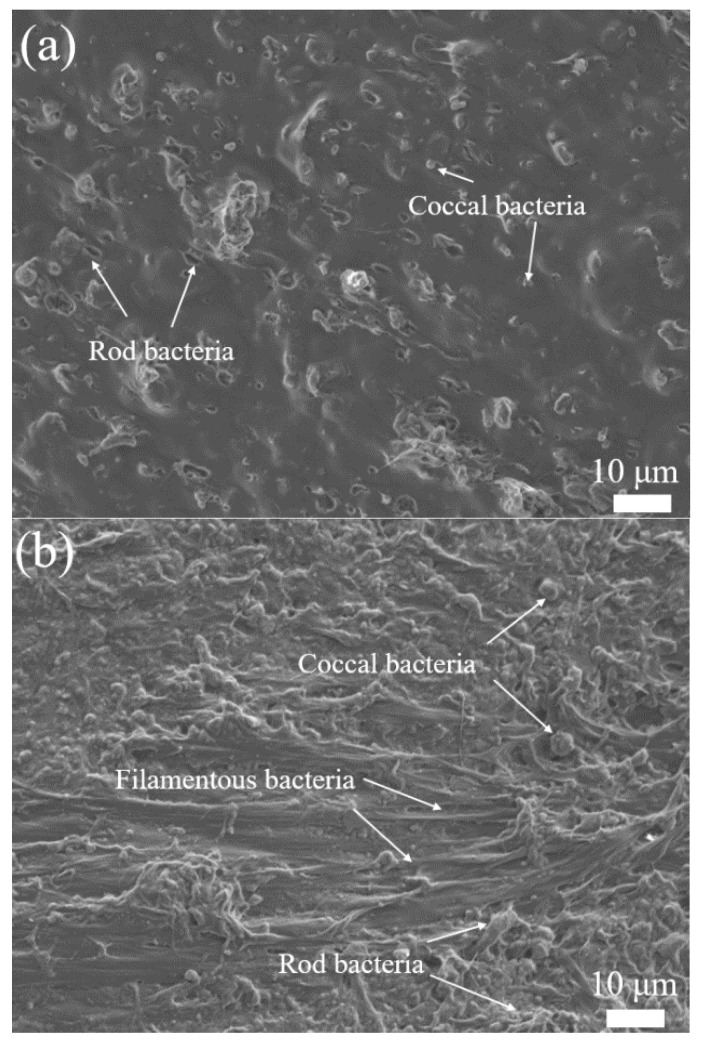
SEM of biofilm attached to carriers: (**a**) Naked-carrier, (**b**) Carrier-PLA-cavity.

From SEM observation, the structure of the biofilm attached to the naked-carrier and carrier-PLA-cavity appeared to be significantly different (Figure 4). The biofilm of the naked-carrier contained coccal and short rod bacteria (Figure 4a). The carrier-PLA-cavity biofilm comprised predominately filamentous bacteria as structural support, coccal, and rod bacteria interspersed into the support (Figure 4b), while mildew fungi were more plentiful, with some short rod bacteria in the naked-carrier biofilm. In addition, the bacteria were surrounded by extracellular polymers, which combined to form a whole connected biofilm structure, increasing the contact area between the biofilm and substrate. Chu et al. [1] illustrated that microorganisms only adhered and grew on the surface of carriers, leading to low biomass and weak attachment. Guillaume et al. [31] and Grumezescu et al. [24] reported the fabrication of PLA-PCL or PLA-PVA microspheres with several layers that showed excellent properties in terms of microorganism adhesion and biofilm formation.

**Figure 5:**
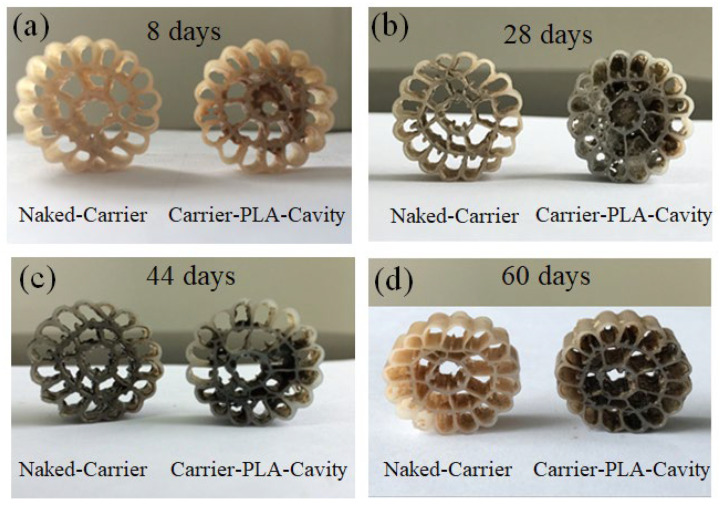
Growth trend of biofilms of Naked-carrier and Carrier-PLA-Cavity.

Few studies have focused on the growth stages of biofilms of solid carbon source carriers, which are crucial to understanding the impact of biofilms on wastewater treatment [14,28,32,33,34]. Figure 5 shows the growth trend of bacterial biofilms on carrier-PLA-cavity and naked-carrier.

The process of the colonization experiment for the development of biofilm is described as four successive periods, namely, the incubation period (stage I: 0–10 days), the growth and mature period (stage II: 10–30 days), the aging period (stage III: 30–45 days), and the next cycle growth period (stage IV: 45–60 days). In stage I, the carrier is slowly inoculated by microorganisms, resulting in low biofilm growth. By stage II, after inoculation by the activated sludge in the reactor, biofilms grew faster with a higher specific surface area of the carrier. Figure 3 shows that carrier-PLA-cavity, with more roughness, has more biomass than the naked-carrier. This continues until stage III, and the biofilm is stable during this period until shedding. After approximately 40 days, the next biofilm growth cycle begins. To facilitate the analysis of the wastewater performance trend, the thickness and mass were used to characterize the growth process of biofilms on carriers.

**Figure 6:**
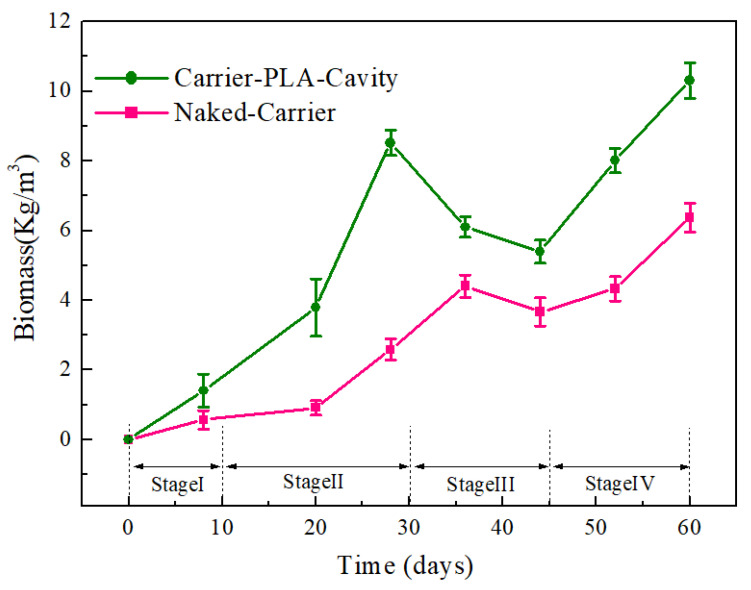
Biomass formed on the different carriers: Carrier-PLA-Cavity and Naked-Carrier, respectively.

The biofilm attached to the carrier-PLA-cavity was thicker than that attached to the naked-carrier (Figure 6). This indicates that the biofilm growth speed of carrier-PLA-cavity is faster than that of the naked-carrier.

The biomass value of carrier-PLA-cavity was three times higher than that of the naked-carrier at day 30. This can be attributed to the larger specific surface area and greater compatibility of carrier-PLA-cavity, leading to shortened growth and maturation periods. After 40 days, the biofilm was removed from the carrier. Since the PLA film/matrix is more easily hydrolyzed than the PE matrix in wastewater, the aging time of the carrier-PLA-cavity biofilm was longer than that of the naked-carrier biofilm, which serves the purpose of updating the biofilm. If the biofilm could not be self-renewed, the aging biofilm would accumulate, leading to a decline in the water quality. The next cycle of biofilm growth began at approximately 44 days. The biomass value of carrier-PLA-cavity was higher than that of the naked-carrier. The highest biomass values of carrier-PLA-cavity and naked-carrier were 10 and 6.5 kg/m^3^ at 60 days, respectively. This indicates the more suitable adhesion of microorganisms on the carrier-PLA-cavity compared to the naked-carrier for the new cycle of biofilm growth, which follows our hypothesis.

Biofilm thickness is a principal parameter used to evaluate the substrate consumption rate in biofilms [35]. According to Chu et al. [1], because the substrates (e.g., carbon and nitrogen sources) crossed the biofilm–liquid interface and were transported through the biofilm to reach the microbial cells and be consumed, there was a limited biofilm thickness.

**Figure 7:**
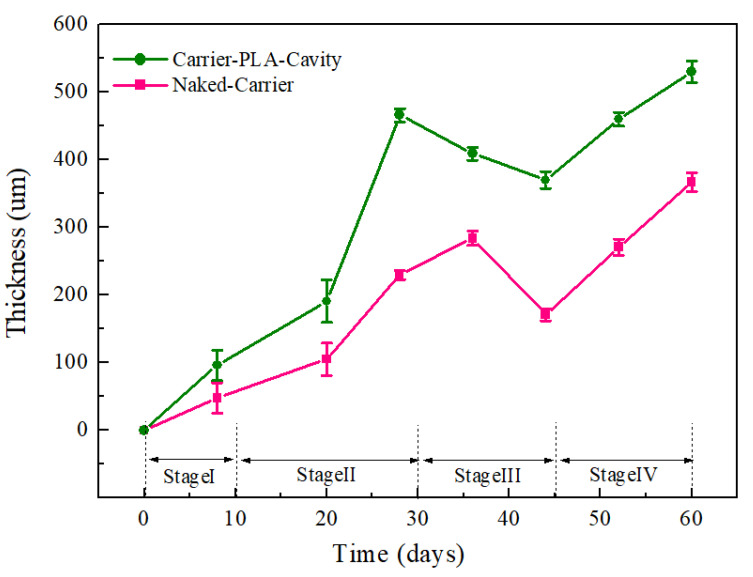
The thickness of biofilms attached to different carriers: Carrier-PLA-Cavity and Naked-Carrier, respectively.

In this study, the increased thickness of the biofilm limited the mass transfer of substrate, resulting in the maximum biofilm thicknesses of carrier-PLA-cavity and naked-carrier being 500 and 350 μm at 60 days, respectively (Figure 7).

Moreover, the higher specific surface area of carriers enabled the ability to retain more microbes from the inoculated sludge, resulting in a higher biofilm thickness [36]. Since carrier-PLA-cavity is etched by NaOH, it has a higher specific surface area than the naked-carrier. For the naked-carrier, the biofilm mainly accumulated in the biocarrier interspaces. However, the biofilm of carrier-PLA-cavity existed not only inside the carrier but also on the surface of the carrier. Therefore, the biofilm thickness of carrier-PLA-cavity was higher than that of the naked-carrier (Figure 7). This indicates that carrier-PLA-cavity can offer certain advantages for biofilm growth by providing an appropriate microenvironment for microbe growth in wastewater treatment.

### 3.3. Wastewater Treatment Performance of Bio-Carriers

**Figure 8:**
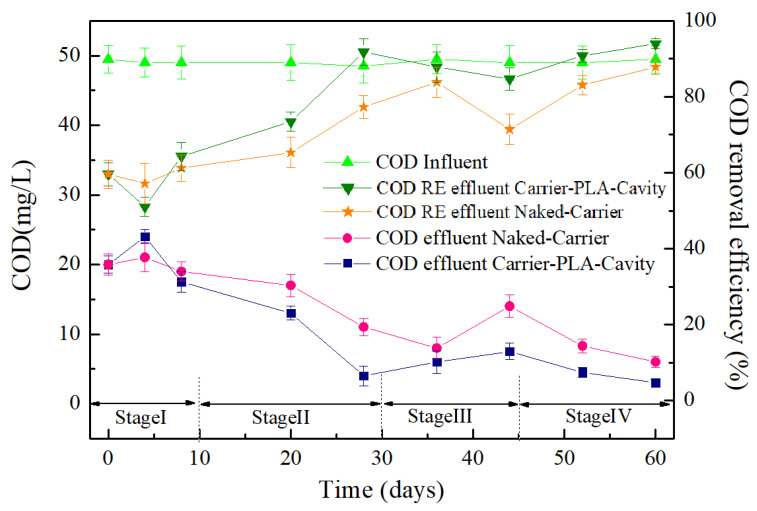
COD removal efficiency (RE) for Carrier-PLA-Cavity and Naked-Carrier.

The COD values were measured over the operating life from 4 days to 60 days, when the biofilm was considered changed from inoculation to mature and stable periods (Figure 8).

Initially, the effluent COD was decreased by the residual activated sludge inside the carriers. After 4 days, the effluent COD of carrier-PLA-cavity increased greatly and then decreased. The decomposition of PLA released organics as a carbon source, which promoted the growth of microorganisms. In turn, microbes consumed organics, resulting in a decrease in effluent COD. In stage II, the COD removal efficiency began to increase rapidly. This is mainly dependent on the growth of biofilms, which consume more organics. This indirectly indicates that carrier-PLA-cavity has better biological activity than the naked-carrier. In stage III, the shedding of biofilm led to the decline in COD removal efficiency. It is interesting that the next growth cycle of biofilms was observed in this study. With the secondary growth of biofilm, COD removal efficiency was further promoted to 94% for carrier-PLA-cavity and 86% for the naked-carrier.

**Figure 9:**
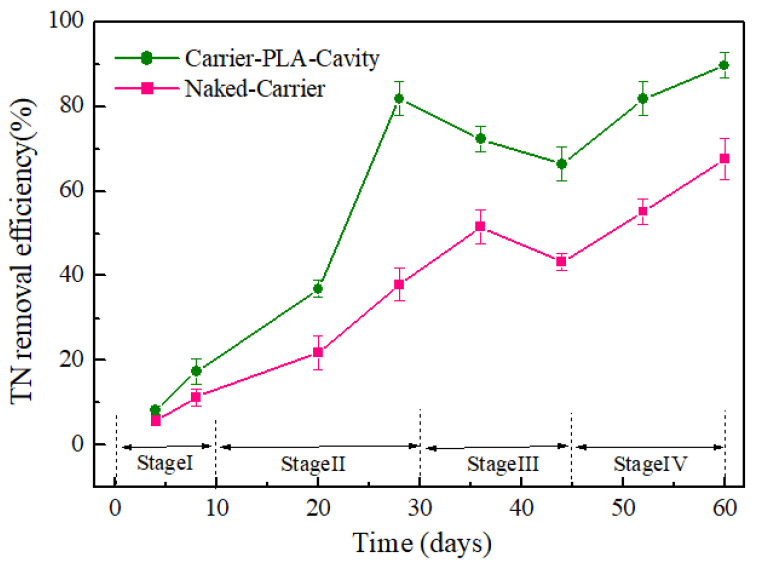
TN removal efficiency for Carrier-PLA-Cavity and Naked-Carrier.

The removal of TN, i.e., NH_4_-N, NO_3_^−^-N, and NO_2_-N, has always been the main index that restricts the standard discharge of low-C/N-ratio wastewater treatment. The TN removal rates were relatively low during the initial phase of sequence batch operation; with increasing sequence time, more rapid TN removal was observed (Figure 9). At the end of stage III, the removal efficiencies of TN of carrier-PLA-cavity and naked-carrier were 66% and 43%, respectively. In stage IV, it was finally increased to 90% and 68%, respectively. Further studies on nitrification and denitrification of the carriers are needed to explain this phenomenon.

### 3.4. Microbial Community Analysis

The bacterial community of the carrier biofilms was analyzed for the number of operational taxonomic units (OTUs), rarefaction, and species richness (Table 5). The numbers of OTUs were estimated at 97% 16S rRNA gene sequence similarity. In the two samples, the OTUs were 195 (carrier-PLA-cavity) and 192 (naked-carrier), the Shannon index was 2.427 and 2.496, and the Chao value was 206 and 222.

**Figure 10:**
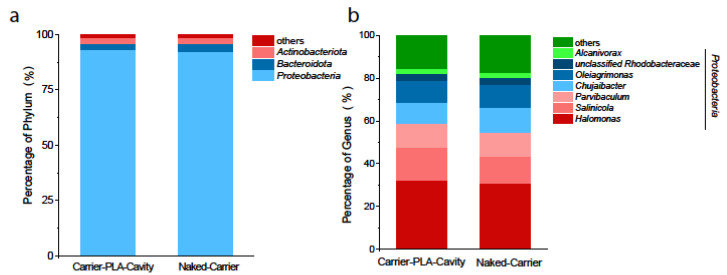
Bacterial community composition at phylum level (**a**) and genus level (**b**).

The community structure of each sample was analyzed at all levels (domain, kingdom, phylum, class, order, family, genus, and species). In this paper, the phylum- and genus-level taxonomic distributions are mainly discussed. Among the trimmed sequences, a total of 20 phyla and 178 genera were identified in the biofilm samples. As shown in Figure 10a, at the phylum level, *Proteobacteria* (96.46%, 93.9%), *Bacteroidota* (3.84%, 5.12%), and *Actinobacteriota* (4.48%, 4.48%) were the three most abundant phyla in all samples. At the genus level, the results (Figure 10b) showed that the fabricated carrier exerted little influence on the microbial community structure compared with the original carrier. *Halomonas* (32%, 30%), *Salinicola* (15%, 14%), *Parvibaculum* (11%, 11%), *Chujaibacter* (10%, 12%), and *Oleiagrimonas* (9%, 10%) were dominant bacteria in both reactors. These bacteria are all heterotrophic and show tolerance to salinity [37], which might be an important reason for their enrichment in the reactor. *Halomonas* was the most abundant genus and is related to the removal of nitrogen [12], suggesting that heterotrophic nitrification and denitrification significantly contributed to the removal of ammonium and nitrate in both reactors. In contrast, autotrophic nitrifiers were not found in the carrier biofilm and further indicated that heterotrophic nitrification was responsible for ammonium oxidation.

### 3.5. Degradation of Carrier-PLA-Cavity and Denitrification

**Figure 11:**
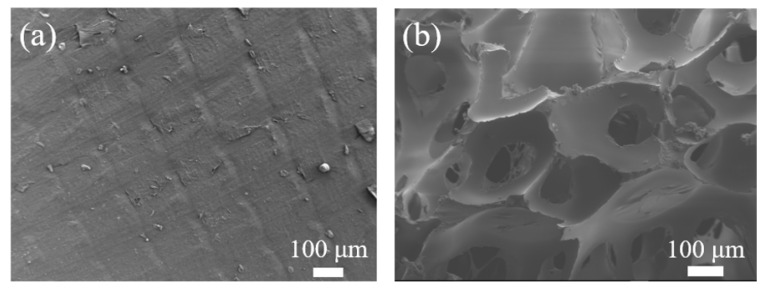
SEM of used carriers: (**a**) Naked-carrier, (**b**) Carrier-PLA-cavity.

Simultaneous nitrification and denitrification (SND) can be used to describe the nitrification and denitrification of biofilms [25]. The biofilm is the site where SND occurs. According to the microbial community data, nitrification and denitrification were all accomplished by heterotrophic bacteria in both reactors. Despite the assimilation of ammonium and nitrate, heterotrophic nitrifiers converted NH_4_^+^ to NO_2_^−^ or NO_3_^−^, and then NO_2_^−^ or NO_3_^−^ was reduced by heterotrophic denitrifiers in the traditional way (NO_3_^−^→NO_2_^−^→NO→N_2_O→N_2_↑). Therefore, a certain amount of carbon is necessary to denitrify the nitrite and nitrate that are formed during nitrification. Comparing the SEM images of the used carriers (Figure 11) revealed that the surface of the naked-carrier was flat and smooth, whereas the surface of the carrier-PLA-cavity was pitted and perforated by microbial degradation. This indicates that carrier-PLA-cavity can provide a carbon source for denitrification, which is conducive to the removal of TN.

**Figure 12:**
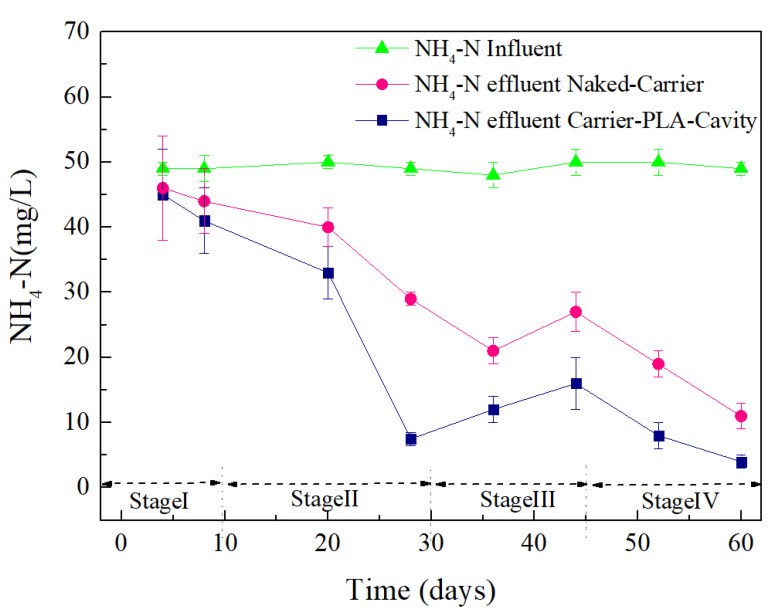
NH_4_^+^-N removal effect for Carrier-PLA-Cavity and Naked-Carrier.

**Figure 13:**
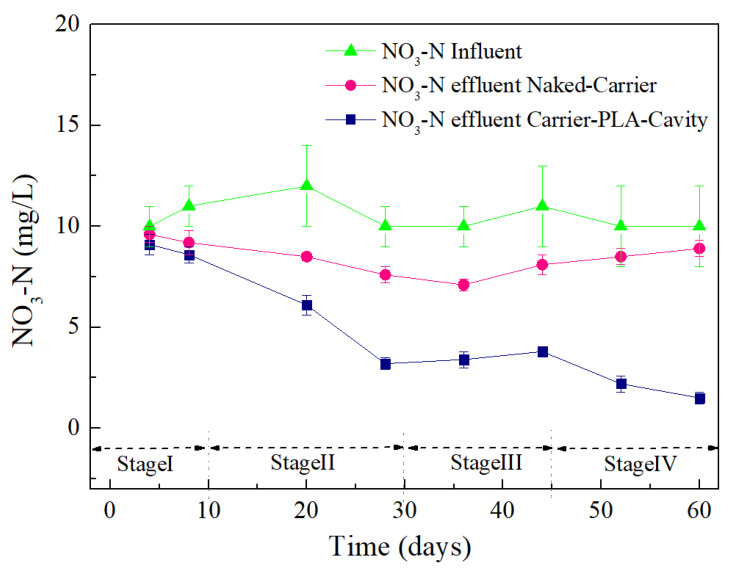
NO_3_^−^-N removal effect for Carrier-PLA-Cavity and Naked-Carrier.

In general, for the most readily available organic carbon source, a COD/NO_3_^−^-N ratio from 3.0 to 6.0 enables complete nitrate reduction to nitrogen gas [25]. To clarify the nitrification and denitrification of different carriers at a low COD/NO_3_^−^-N ratio (approximately 0.83), NH_4_^+^-N, NO_3_^−^-N, and NO_2_^−^-N of carriers were monitored, as shown in Figure 12 and Figure 13. The effluent NO_2_^−^-N remained at a low level (less than 0.9 mgL^−1^) throughout the experiments, indicating that nitrite oxidation took place almost completely.

NH_4_^+^-N gradually decreases with the progress of the reaction, but the removal rate of NH_4_^+^-N by the naked-carrier is slower than that of carrier-PLA-cavity (Figure 12). Due to the loose and porous structure of carrier-PLA-cavity, this substrate is more conducive to the transport of oxygen and increases the ammonium oxidation rate.

Considering the very low COD/TN ratio in the influent, it is apparent that the biodegradable polymer PLA was responsible for the denitrified nitrate in the reactor. Shen et al. studied blends of cross-linked starch/polycaprolactone biocarriers and showed a NO_3_^−^-N removal efficiency greater than 90% when adding a sufficient carbon source [22]. Chu et al. compared the NO_3_^−^-N removal efficiency of the biocarrier with and without glucose and showed that denitrification was greatly stimulated by glucose. In this study, the removal of NO_3_^−^-N from the treated carrier was gradually reduced as the reaction progressed (Figure 13). However, the NO_3_^−^-N of the naked-carriers decreased first and then gradually increased and accumulated. This is due to the lack of carbon sources, which prevents denitrification.

The rSND could be defined as follows by neglecting the effect of assimilation and cellular decay on ammonium during the experiments [25]:

(1) $$\mathrm{rSND}=\left(1-\frac{{\left[{\mathrm{NO}}_{X}N\right]}_{\mathrm{remained}}}{{\left[{\mathrm{NH}}_{4}N\right]}_{\mathrm{removed}}}\right)\times 100\%$$

**Figure 14:**
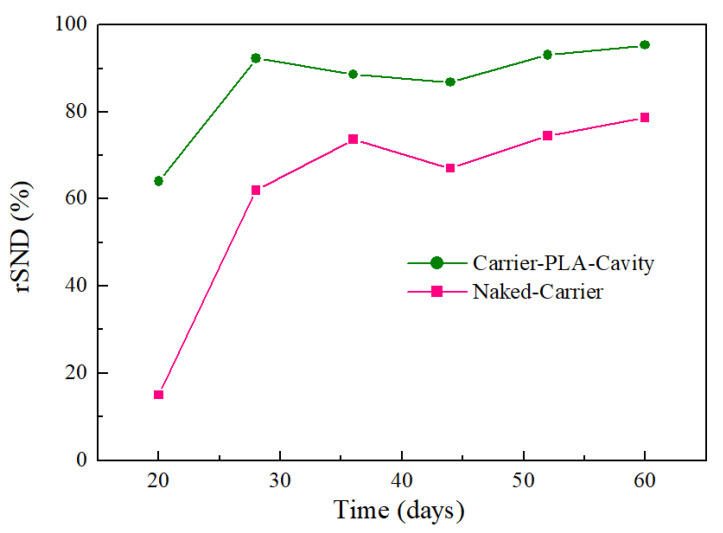
The rSND for Carrier-PLA-Cavity and Naked-Carrier during the reaction.

Compared with the naked-carrier, carrier-PLA-cavity has higher rSND values (Figure 14), indicating that it can remove more NH_4_^+^-N and retain less NO_x_-N. This is because PLA can be used as a solid carbon source and release carbon through gradual hydrolysis, providing power for denitrification. At a low C/N ratio, the naked-carrier is unable to digest NO_x_-N due to its low denitrification, leading to gradual accumulation [25]. In addition, this also explains why carrier-PLA-cavity can achieve a higher TN removal rate, because it can effectively reduce NO_x_-N through denitrification.

## 4. Conclusions

In this research, a novel two-step coating–etching method was used to fabricate biocarriers with a high specific surface area and biodegradability. By monitoring the biofilm growth cycle, it was found that the biofilm on carrier-PLA-cavity grew and updated faster than the naked-carrier. The biomass and thickness of biofilms growing on carrier-PLA-cavity were higher than those on the naked-carrier, indicating that the bioactivity of the biofilm attached to carrier-PLA-cavity was higher. The COD and TN removal efficiency values of the carrier-PLA-cavity were higher than those of the naked-carrier. The rSND monitoring results showed that carrier-PLA-cavity dominated denitrification to consume NO_x_-N. The microbial community analysis of the carrier biofilms showed that *Halomonas* was the most abundant genus, and heterotrophic nitrification and denitrification were responsible for nitrogen removal in both reactors. These results demonstrate that the low cost and facilitation of the coating–etching process for depositing BDP films with microporous cavities on substrates provides a good strategy for the fabrication of biocarriers for treating wastewater with a low C/N ratio.

## Figures and Tables

**Table 1:** The physical and chemical properties of materials.

Materials	Melting Point (°C)	Boiling Point (°C)	Density (g/cm^3^)	Tensile Strength (MPa)	Elongation (%)	Form
K3	105	-	0.96	15	90	Particle
PLA	170	-	1.25	55	5	Powder
NaOH	318	1388	2.13	-	-	Powder
CH_2_Cl_2_	−97	39	1.32	-	-	Liquid

**Table 2:** The composition and content of the trace element solution.

Component	Dosage (g/L)
EDTA	15.000
ZnSO_4_·7H_2_O	0.430
CoCl_2_·6H_2_O	0.240
MnCl_2_·4H_2_O	0.990
CuSO_4_·5H_2_O	0.250
NaMoO_4_·2H_2_O	0.220
NiCl_2_·2H_2_O	0.190
Na_2_SeO_4_·10H_2_O	0.210
H_3_BO_4_	0.014
Na_2_WO_4_·2H_2_O	0.050

**Table 3:** The composition and content of the mineral medium.

Component	Dosage (g/L)
NaH_2_PO_4_·2H_2_O	0.029
CaCl_2_·2H_2_O	0.300
MgSO_4_·7H_2_O	0.200
FeSO_4_·7H_2_O	0.00625
EDTA	0.00625

**Table 4:** The parameters of synthetic wastewater.

Component	Dosage
COD in influent	50 mg/L
NH_4_^+^-N in influent	50 mg/L
NO_3_^−^-N in influent	10 mg/L
NaCl in influent	30 g/L
pH	7.0~8.2
Trace elements	1.25 mL/L
Mineral medium	As Table 2 showed

**Table 5:** The sequence information and diversity index.

Sample	Sequence Number	OUT	Alpha Diversity Index				
			Ace	Chao	Shannon	Simpson	Coverage
Carrier-PLA-Cavity	53,617	195	212	206	2.427	0.163	0.999
Naked-Carrier	58,259	192	223	222	2.496	0.151	0.999

## Data Availability

Data available upon request due to privacy and ethical restrictions.
